# Loss of Cytotoxicity and Gain of Cytokine Production in Murine Tumor-Activated NK Cells

**DOI:** 10.1371/journal.pone.0102793

**Published:** 2014-08-07

**Authors:** Jürgen R. Müller, Thomas A. Waldmann, Sigrid Dubois

**Affiliations:** Metabolism Branch, Center for Cancer Research, National Cancer Institute, National Institutes of Health, Bethesda, Maryland, United States of America; Rutgers - New Jersey Medical School, United States of America

## Abstract

NK cells are able to form a functional memory suggesting that some NK cells are surviving the activation process. We hypothesized that NK cell activation causes the development of a distinct NK cell subset and studied the fate of murine post-activation NK cells. Activation was achieved by in vivo and in vitro exposures to the melanoma tumor cell line B16 that was followed by differentiation in IL-2. When compared with control NK cells, post-activation CD25^+^ NK cells expressed little granzyme B or perforin and had low lysis activity. Post-activation NK cells expressed CD27, CD90, CD127, and were low for CD11b suggesting that tumor-induced activation is restricted to an early NK cell subset. Activation of NK cells led to decreases of CD16, CD11c and increases of CD62L and the IL-18 receptor. In vivo activated but not control NK cells expressed a variety of cytokines that included IFNγ, TNFα, GM-CSF and IL-10. These data suggest that the exposure of a subset of peripheral NK cells to the B16 tumor environment caused an exhaustion of their cytolytic capacity but also a gain in their ability to produce cytokines.

## Introduction

NK cells were identified by their capacity for spontaneous lysis of tumor cells [1]. They express unique subsets of non-polymorphic NK cell receptors (NKRs) that deliver either activating or inhibiting signals [2]. NK cells are constantly scrutinizing somatic cells for their expression of NKR ligands. Both an increased surface expression of ligands for activating receptors as observed under stress conditions and decreases of inhibitory receptor ligands found during transformation and viral infections signal to NK cells to lyse affected cells.

The formation of NK cell-mediated memory has been described in the murine CMV model [3]. This suggests that some NK cells must survive their initial activation to form memory cells. It would therefore be of interest to monitor changes in NK cells that are induced by their activation. Our hypothesis is that post-activation NK cells would form a functional and/or phenotypical distinct NK cell subpopulation.

Murine NK cells can be divided based on their expression of CD27 and CD11b [4]. Hayakawa and Smyth reported subsets based on their CD27 expression that showed some characteristics of CD56^br**ight**^ NK cells [5]. Murine CD27^high^ and CD27^low^ NK cells differed in their cytotoxicity, cytokine production and tissue distribution. The CD27^low^ subset was excluded from murine lymph nodes.

Subsets of human NK cells differ from mice and are mainly defined by their surface expression of NCAM (CD56) [6]–[10]. While CD56^dim^ NK cells have high cytolytic potential, CD56^br**ight**^ NK cells largely lack granzyme B and perforin resulting in low cytotoxicity [11]–[13]. CD56^br**ight**^ but not CD56^dim^ NK cells have a high capacity to produce a variety of cytokines suggesting immune-regulatory functions of CD56^br**ight**^ NK cells. Both NK cell subsets have distinct expression patterns of surface markers such as CD25, CD16 and CD62L [7], [8], [14]. Both NK cell subsets also differ in their tissue distributions. CD56^br**ight**^ NK cells are predominantly found in lymph nodes [15] and are the predominant NK cell type in placental tissue suggesting a role during pregnancy [16]. CD56^br**ight**^ NK cells may also be negatively involved in autoimmune disease as their number has been inversely correlated with clinical severity of multiple sclerosis [17], [18]. It is currently unknown whether CD56^dim^ and CD56^br**ight**^ NK cells represent functionally distinct subsets that are derived from a common precursor, or whether they represent different NK cell maturation stages.

It has been noted that subsets of human NK cells differ in their NKR expression patterns [13]. In particular, NK cells that are negative for inhibitory killer cell immunoglobulin-like receptors (KIRs) are predominantly found within the CD56^dim^ subset. Several groups have defined NK cells without inhibitory KIR expression as “unlicensed” or “hypo-responsive” to stimuli such as missing MHC class I expression [19]–[21]. The involvement of an activation step in the development of CD56^br**ight**^ NK cells would be consistent with the lack of KIR-negative cells in this subset since the low likelihood of activation within this “hypo-responsive” group would largely prevent their differentiation into CD56^br**ight**^ NK cells. It has also been proposed that CD56^br**ight**^ NK cells represent recently activated CD56^dim^ NK cells [22]. This is supported by the presence of CD56^br**ight**^ NK cells at sites of inflammation [23], [24].

Here we describe functional and phenotypical changes in murine NK cells that are induced by an in vivo exposure to tumor environment. We show that recently activated NK cells loose their cytotoxicity, produce cytokines and modulate some surface marker expressions.

## Results

### Activation of NK cells is supported by IL-15

We were interested in studying post-activation NK cells. Activation as measured by the expression of CD25, CD69 or IFNγ can be achieved by Fc receptor cross-linking, exposure to NK-sensitive tumor cells, co-incubation with mature dendritic cells, in vivo infections with bacteria such as listeria monocytogenes, among others ([1], [25]–[27] and data not shown). We choose as a model system the activation by the NK-sensitive melanoma line B16. Activation of NK cells by this tumor cell line appears to be caused by a classical “missing self” recognition since both activation and NK cell cytotoxicity are greatly reduced by IFNγ–induced MHC class I up-regulation on B16 cells prior to exposure to NK cells ([28] and data not shown).

**Figure 1:**
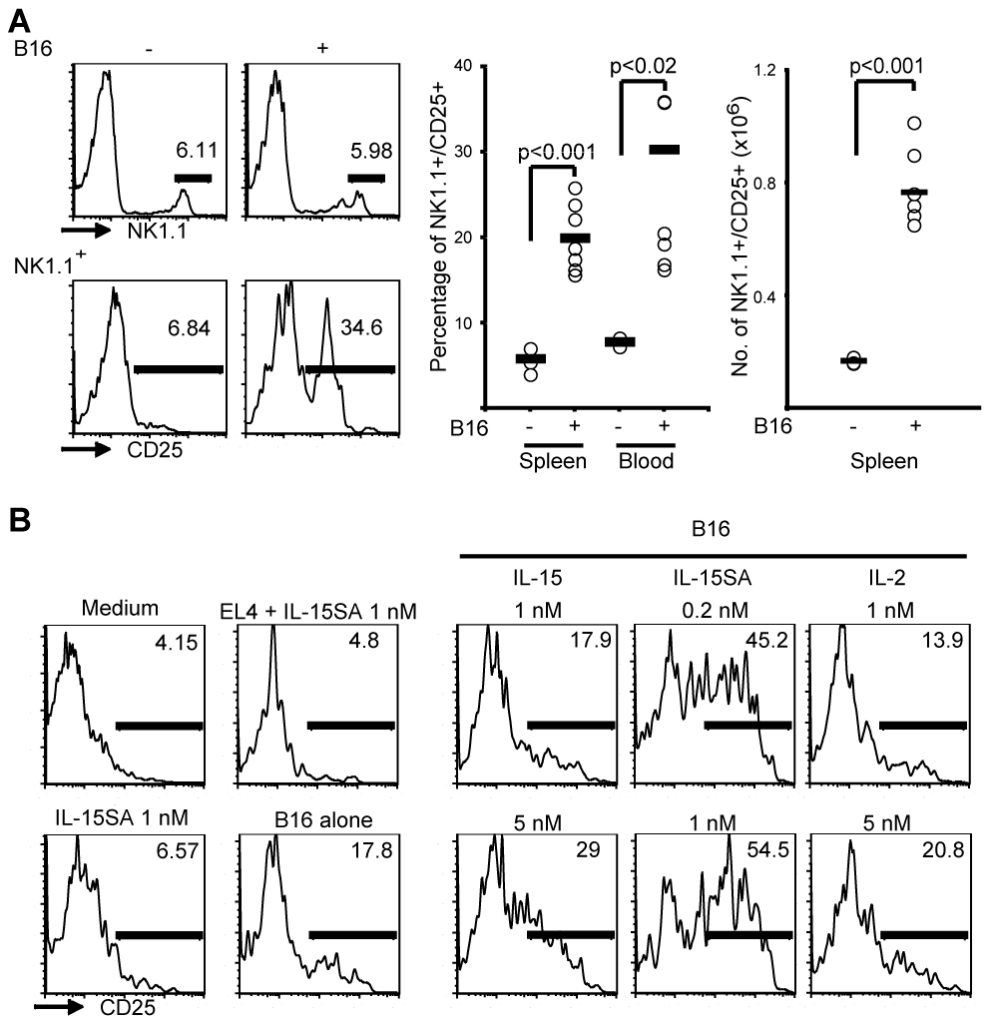
Activation of NK cells after exposure to B16 is supported by IL-15. A. Mice were injected with B16 tumor cells, and the activation status of NK cells was assessed 21 days later. An example (left) shows that while little effect was seen on the percentage of NK1.1^+^ cells among PBMCs (upper panels), a high percentage of NK1.1^+^ cells expressed CD25 only in the presence of tumors (lower). Both the percentage of NK1.1^+^/CD25^+^ cells among splenocytes and PBMCs (middle) and their total numbers in spleens (right) were increased by the presence of B16 tumors. B. IL-15 supports NK cell activation. One million freshly isolated murine NK cells were cultured in medium, in 1 nM IL nM IL-15SA, or were co-cultured with equal numbers of NK-insensitive EL-4 control cells or NK-sensitive B16 cells. Analyzing CD25 among NK1.1^+^ cells 6 h later showed that co h later showed that co-incubation with B16 but not with EL-4 had caused the activation of a small percentage of NK cells. While the presence of 5 nM IL nM IL-15 and of 0.2 or 1 nM IL nM IL-15SA in B16 co-incubation cultures strongly increased this percentage compared with B16 alone at left, IL-2 was only marginally active. Data were reproducible in at least three independent experiments.

In vivo activation of NK cells was achieved by transplanting one million B16 melanoma cells. When mice bore substantial numbers of tumor cells 21 days after transplantation, we observed significant increases in the percentage of blood and spleen NK1.1^+^ cell populations that expressed the activation marker CD25 when compared to untreated mice (Fig. 1A, left and middle). The total number of NK1.1^+^/CD25^+^ cells was also increased in spleens from B16-bearing mice (Fig. 1A, right). To exclude a contribution of NK1.1^+^-expressing B or T lymphocytes, the experiment was repeated in *Rag1*-deficient mice giving similar results (data not shown). This suggests that the presence of B16 tumor cells caused NK cell activation in vivo independently of B or T lymphocytes.

To analyze activation in vitro, splenic NK cells were isolated from untreated mice by negative sorting that resulted in more than 95 % purity (not shown). Incubations in medium alone or with 1 nM IL-15 super-agonist (IL-15SA, comprising murine IL-15 bound to a murine IL-15Rα/human IgG1-Fc hybrid protein) as well as with equal numbers of NK-insensitive EL-4 cells for 6 hours caused little expression of CD25 on NK cells (Fig. 1B). In contrast, after co-incubations with equal numbers of B16 cells, a small percentage of NK cells had acquired an activated phenotype within 6 hours. The percentage of NK cells that expressed CD25 was increased if this co-culture was done in the presence of nanomolar concentrations of IL-15 or of picomolar concentrations of IL-15SA [29]. The presence of IL-2 at nanomolar concentrations had little effect on NK cell activation. These data suggest that maximum tumor-induced NK cell activation requires an interaction with target cells and signaling induced by IL-15.

### Reduced cytotoxicity in post-activation NK cells

Prolonged in vitro exposure to tumor target cells (24 h) causes apoptosis of most NK cells (not shown). However, interactions between NK cells and their targets may be more limited in vivo to allow for the survival of some NK cells that establish memory [3]. To follow their post-activation fate, we activated NK cells in vivo by transplanting B16 into mice. The resulting CD25^+^ NK cells were analyzed ex vivo and compared with CD25^−^ NK cells from the same mice and with NK cells from naïve mice. To allow for post-activation differentiation, NK cells were also sorted, and the resulting CD25^−^ and CD25^+^ NK cells were expanded in vitro in IL-2 for 7 days.

**Figure 2:**
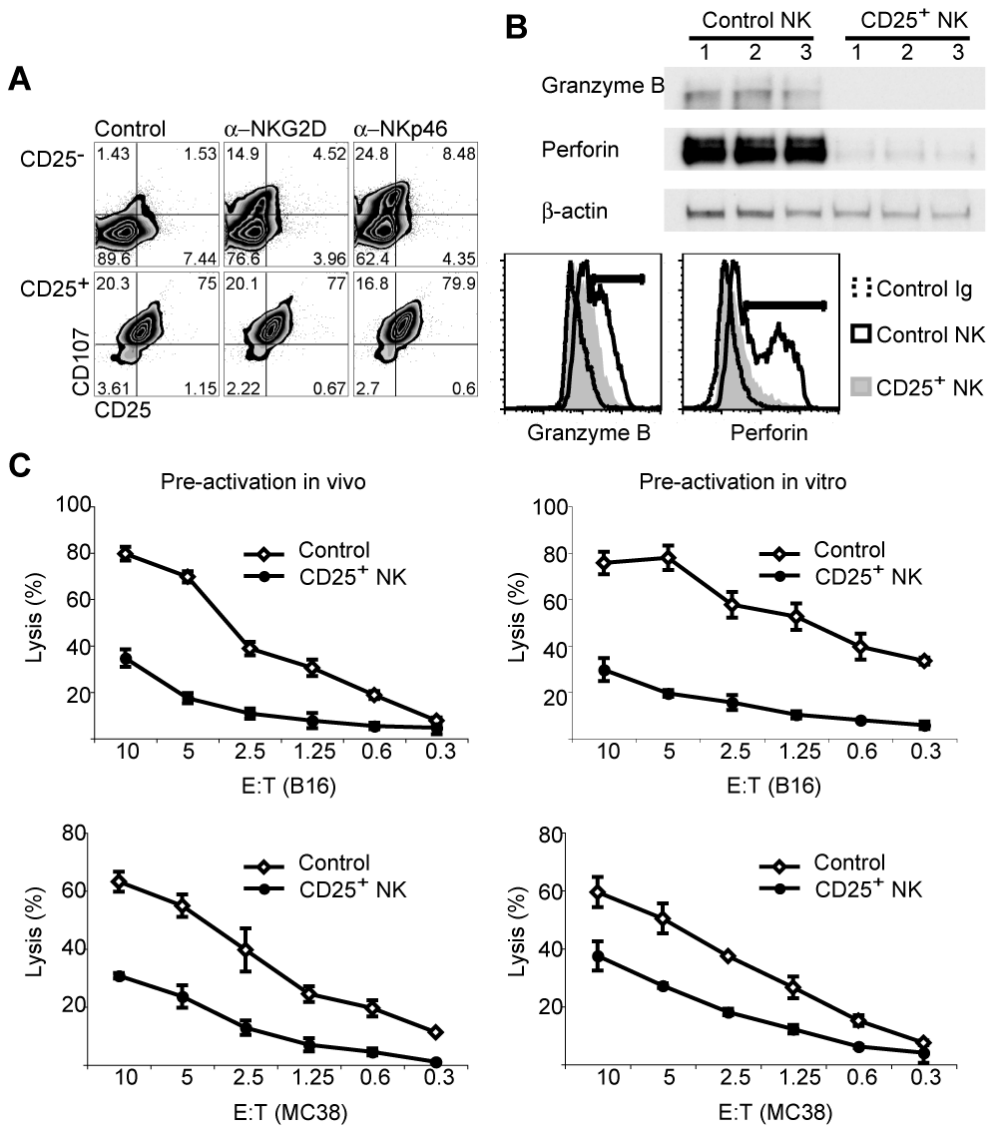
Post-activation NK cells have reduced cytolytic activity. A. Post-activation CD25^+^ and non-activated CD25^−^ NK cells were isolated from spleens of B16-bearing mice and were incubated immediately with activating antibodies against NKG2D or NKp46 in the presence of anti-CD107 antibody to detect degranulation. While the degranulation had to be induced in CD25^−^ NK cells by the activating antibodies (top row), CD25^+^ NK cells degranulated spontaneously. B. Post-activation CD25^+^ NK cells isolated from spleens of B16-bearing mice and control NK cells isolated from normal spleens were expanded for 7 days in IL-2 in vitro. Western analyses (top) using three independent isolations each revealed reduced protein levels of granzyme B and perforin but similar levels of β-actin in post-activation NK cells. Intracellular cytometry analyses (bottom) of NK1.1^+^ cells showed that only control but not post-activation NK cells contained populations with high levels of granzyme B and perforin. C. NK cells that had been activated in vivo in B16-bearing mice (left) or in vitro (right) and control NK cells were sorted and expanded in IL-2. Lysis assays revealed reduced cytotoxicity of post-activation NK cells both towards B16 (upper) and towards MC38 cells (lower) when compared to control NK cells. Data are shown +/− SD. Analyses were done in at least three independent experiments giving similar results.

Ex vivo analyses revealed that CD25^+^ NK cells spontaneously degranulated as measured by CD107 expression under control conditions (Fig. 2A). A small increase in CD107 intensity was observed after activating these post-activation NK cells with antibodies cross-linking NKG2D or NKp46. Degranulation in CD25^−^ NK cells that had been isolated from the same tumor-carrying mice had to be induced by antibody-mediated cross-linking of NKG2D or NKp46 (Fig. 2A). After a 1-week exposure to IL-2, post-activation NK cells when compared with control NK cells had low expression of granzyme B and perforin as revealed by Western blotting and cytometry (Fig. 2B). The same post-activation NK cells had reduced cytolytic activity both towards B16 cells that had been used for activation and towards the unrelated MC38 colon carcinoma cell line that is also recognized by NK cells (Fig. 2C, left). To control for tumor cell-independent effects of the tumor environment, post-activation NK cells were also generated by in vitro exposures to B16 resulting in similar decreases of cytotoxicity and granzyme/perforin expressions (Fig. 2C, right and not shown). These data appear to show that post-activation NK cells are unable to regenerate granzyme B and perforin resulting in reduced cytolytic activity.

### NK cell surface marker expression in post-activation NK cells

Differences in the expression of a number of surface markers have been reported for subsets of murine and human NK cells [4], [7], [8], [14]. We determined whether activation preferentially affects known NK cell subsets, and whether a tumor-induced activation of murine NK cells also causes phenotypical changes either before or after culture in IL-2.

**Figure 3:**
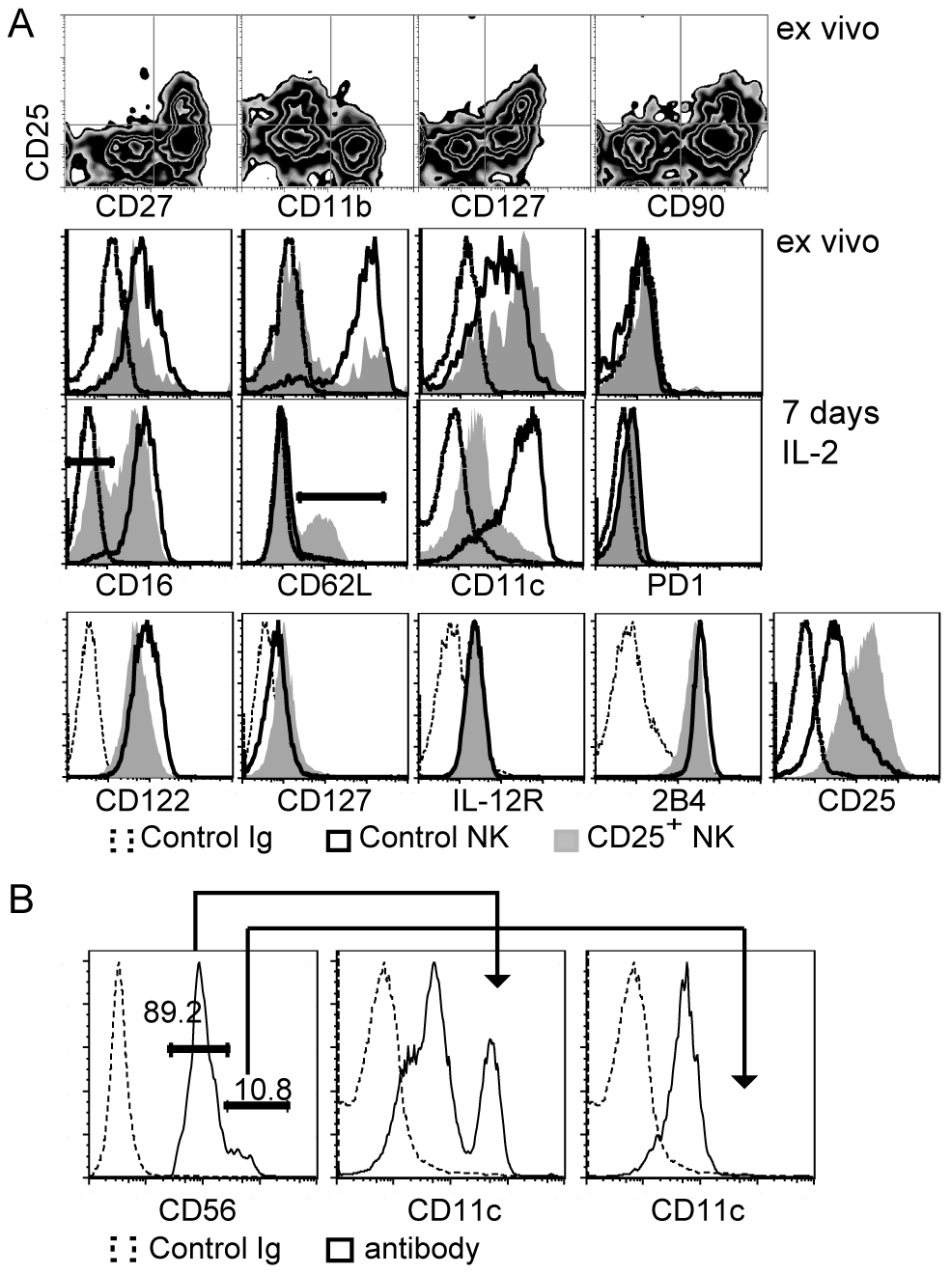
Post-activation and non-activated murine NK cells differ phenotypically. A. Ex vivo analyses of B16-bearing mice revealed that NK cell activation was restricted to a subset that expressed CD27, CD90, CD127 and was low for CD11b (top row). The same cells revealed differences in the expression of CD16, CD62L and CD11c when CD25^+^ were compared with CD25^−^ NK cells (2^nd^ row). Post-activation CD25^+^ NK cells isolated from spleens of B16-bearing mice and control NK cells isolated from normal spleens were also differentiated for 7 days in IL-2 in vitro. Cytometry analyses revealed differences in the surface expression of CD16, CD62L, CD11c and CD25 between both cell types after IL-2 differentiation (3^rd^ row). Little differences were observed for CD122, CD127, IL-12Rβ2 and 2B4 (bottom row). Similar phenotypical differences were seen when CD25^+^ and CD25^−^ NK cells were compared that had been isolated from the same B16-bearing mice and differentiated in IL-2 (not shown). B. Analyses of sorted human NK cells showed that a population with high expression of CD11c is found among CD3^−^/CD56^dim^ but not within the CD3^−^/CD56^br**ight**^ subset of NK cells. All phenotypical differences were reproducible in at least three independent experiments.

Peripheral NK cell differentiation stages in mice are defined by their expressions of CD27 and CD11b [4]. Double-negative immature NK cells transition through CD27-single expression that is followed by double expression and CD11b-single expression in the most mature subset. Analyses ex vivo revealed CD25 expression in the CD27^+^/CD11b^−^ subset suggesting that tumor-induced activation in vivo is restricted to early NK cells. It has also been reported that Foxp3-positive regulatory T cells suppress the expansion of an NK cell subset [30]. Fig. 3A shows that ex vivo CD25 expression was limited to the same CD127^+^/CD90^+^ NK cell population although CD127 expression was lost after in vitro differentiation in IL-2 (see below). These data suggest that tumor-induced NK cell activation is limited to a distinct subset of early NK cells.

We studied whether dynamic changes of additional phenotypical markers are observed as a result of activation. CD16 and CD62L are differently expressed among human NK cell subsets [7], [31]. When analyzed ex vivo after tumor activation in mice, CD25^+^ NK cells had a slightly reduced expression of CD16 compared with CD25^−^ NK cells from the same mice (Fig. 3A) or with NK cells from naïve mice (not shown). A CD16^low^ NK cell population appeared after IL-2 differentiation of CD25^+^ NK cells that was not observed after differentiating CD25^−^ NK cells (Fig. 3A). CD62L expression was down-regulated in post-activation NK cells ex vivo, but CD62L was expressed only in a portion of CD25^+^ NK cells after differentiation. The expression of CD11c has been reported for a sub-population of NK cells [31]–[34]. NK cells that were analyzed after B16-induced activation in vivo (Fig. 3A) or B16/IL-15SA-induced activation in vitro (not shown) had increased CD11c expression when compared with non-activated control cells. This expression pattern markedly changed after culturing both cell populations in IL-2 in that most non-activated NK cells had acquired while most post-activation NK cells had lost CD11c expression (Fig. 3A). Surface expressions that showed little difference between cultured post-activation and non-activated NK cells included subunits of the receptors for IL-2/15 (CD122), IL-7 (CD127) or IL-12 (IL-12Rβ2) as well as the NKR 2B4. CD25 expression differences were retained during IL-2 differentiation while PD1 expression was absent from any NK cells that we analyzed.

Although both CD56^br**ight**^ and CD11c^dim^ human NK cells have been correlated with multiple sclerosis activity [17], [18], [35], differences in the CD11c expression have not been reported for human NK cell subsets. Analyzing PBMCs from normal donors we observed that CD3^−^/CD56^dim^ NK cells contained a sub-population that expressed high amounts of CD11c (Fig. 3B). In contrast, little CD11c expression was observed among CD3^−^/CD56^br**ight**^ NK cells. Together these data show that tumor activation affected a distinct subset of early NK cells that subsequently undergoes dynamic changes in the expression of various surface markers.

### Generation of cytokines by post-activation NK cells

**Figure 4:**
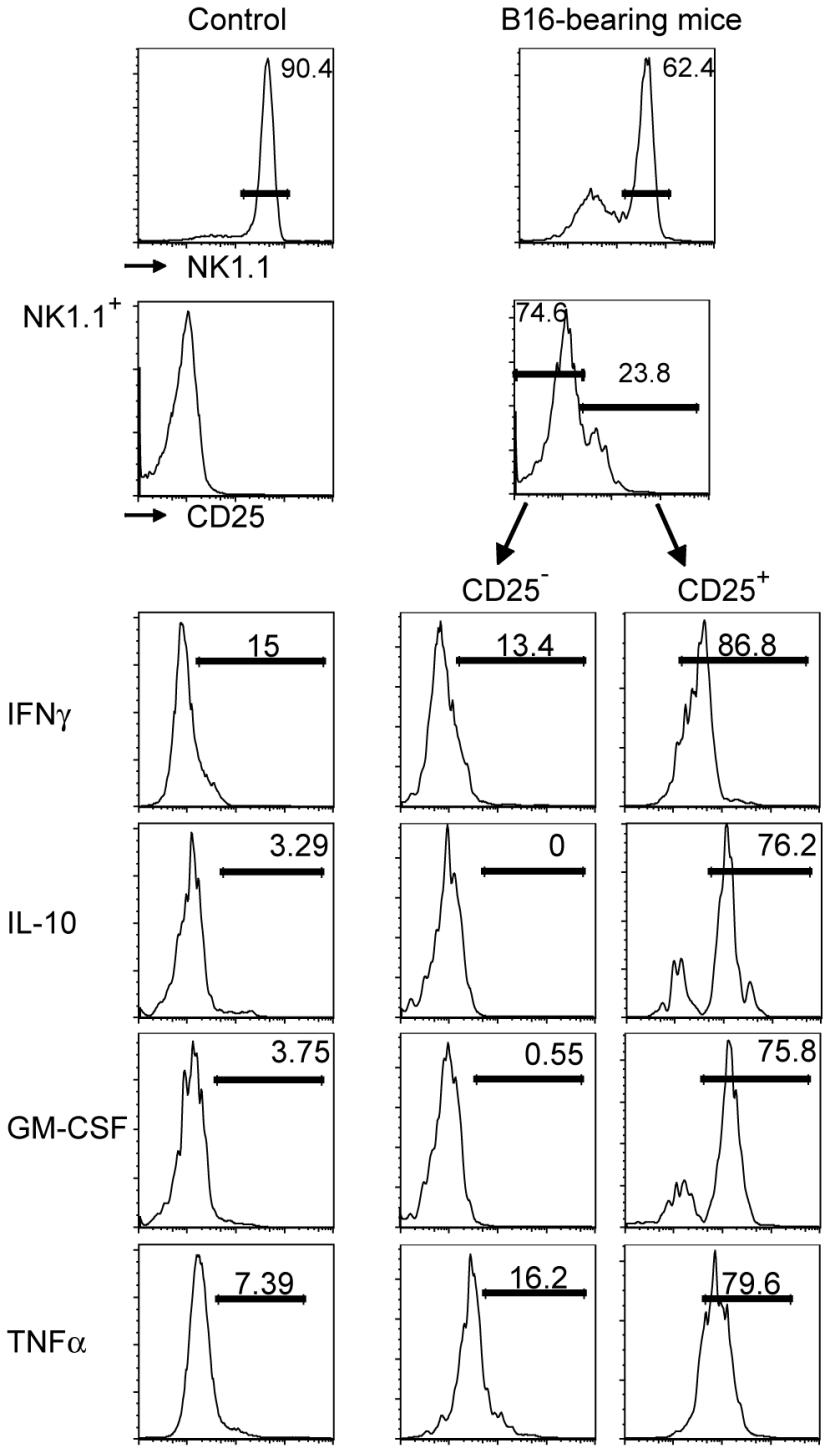
Post-activation NK cells express high levels of cytokines. Spleen cells from control *Rag1^-/-^* mice (left) and from B16-bearing *Rag1^-/-^* mice (right) were incubated for 5 h in brefeldin A and stained for extracellular NK1.1 and CD25 and for intracellular cytokines. Little cytokine expression was observed in control NK cells and in CD25 h in brefeldin A and stained for extracellular NK1.1 and CD25 and for intracellular cytokines. Little cytokine expression was observed in control NK cells and in CD25^−^ NK cells from B16-bearing mice. In contrast, most CD25^+^ NK cells from B16-bearing mice had generated IFNγ, IL-10, GM-CSF and TNFα. Data were reproducible in three independent experiments.

An increased production of various cytokines has been reported for human subsets of NK cells [8]. We studied whether prior activation affects the cytokine production in murine NK cells. Spleen cells from control and from B16-bearing *Rag1* ^-/-^ mice 21 days post tumor transplantation were incubated with brefeldin A for 5 h and analyzed by cytometry. *Rag1* ^-/-^ mice were chosen to avoid contaminations by NK1.1^+^-expressing B or T lymphocytes. We analyzed IFNγ, IL-10, GM-CSF and TNFαthat were chosen based on their generation in human NK cells [36]. Fig. 4 shows that NK1.1^+^ cells from normal mice generated little of the cytokines that were analyzed. Among NK cells from B16 tumor-bearing mice, the majority of the CD25^+^ but few of the CD25^−^ NK cells had generated these cytokines (Fig. 4). One-hour incubations in IL-12, IL-18, PMA + ionomycin and combinations thereof prior to adding brefeldin A failed to increase the number of IFNγ, IL-10, GM-CSF or TNFα-producing NK cells (not shown) suggesting that these NK cells had been fully activated in vivo. No spontaneous or induced cytokine production was observed after IL-2 culture. These data show that a tumor-induced in vivo activation confers onto NK cells the ability to produce various cytokines.

### Induction of the IL-18 receptor by NK cell activation

**Figure 5:**
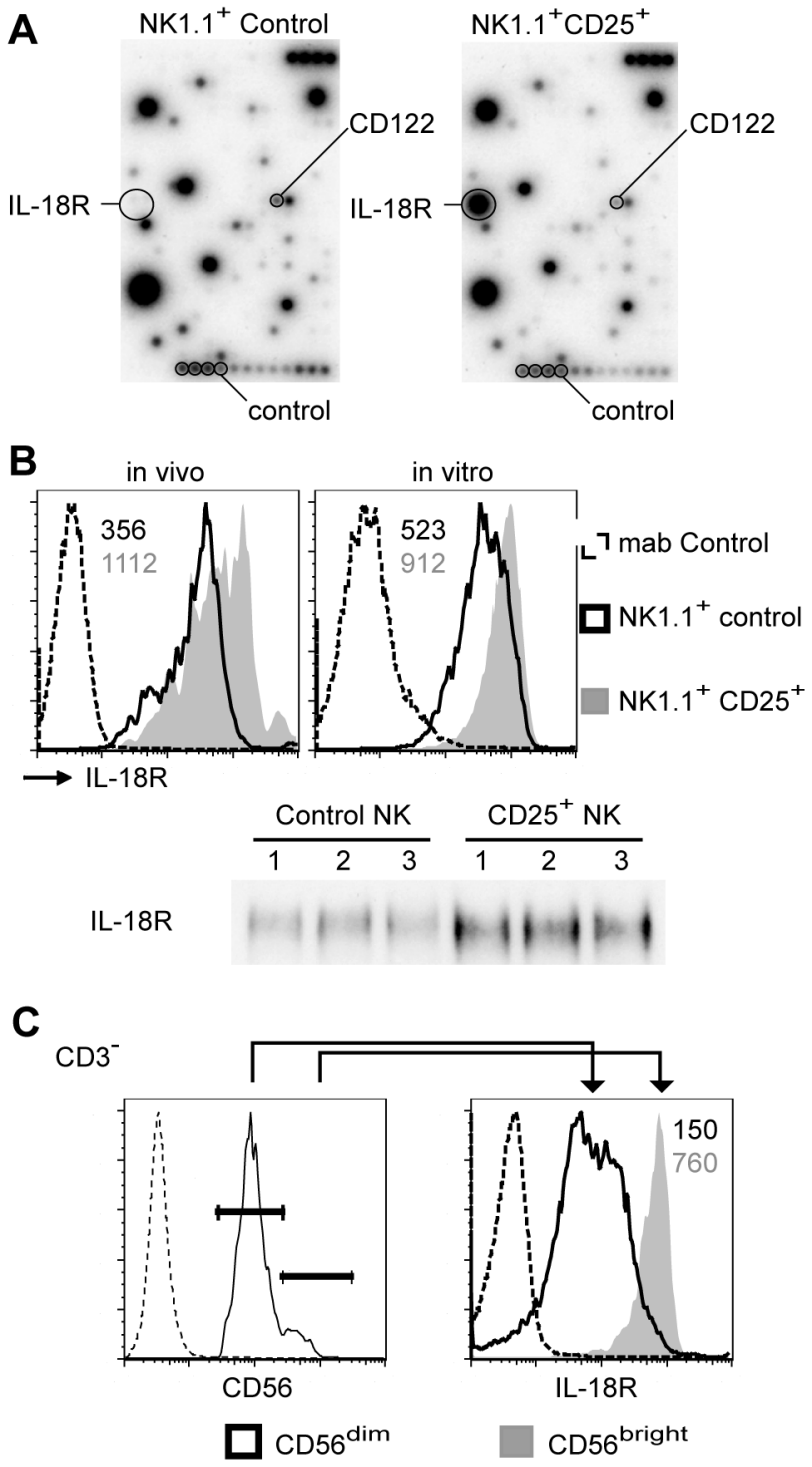
Post-activation NK cells express high levels of the IL-18 receptor. Post-activation CD25-positive and control NK cells were expanded for 7 days in IL-2. An analysis of the expression of various cytokines, receptors and signaling molecules using a microchip revealed an increase of IL-18 receptor RNA levels. B. Cytometry analyses showed increased expressions of the IL-18 receptor in murine CD25^+^ NK cells either from B16-bearing mice (left) or after 6-h incubations with B16 and 0.2 nM IL nM IL-15SA (right) when compared with CD25^−^ control NK cells. Numbers denote mean fluorescence intensities of the IL-18 receptor. An increase of IL-18 receptor protein levels was also observed when in vivo-activated, sorted and IL-2-expanded NK cells were compared with sorted and IL-2-expanded control NK cells by Western blotting. The corresponding β-actin control is shown in Fig. 2. C. Analyses of sorted human NK cells showed higher IL-18 receptor expression in CD3^−^/CD56^br**ight**^ when compared with CD3^−^/CD56^dim^ NK cells. Cytometry analyses were reproducible in three independent experiments.

To find a potential cause for their ability to produce cytokines we subjected RNAs from in vitro-expanded post-activation and control murine NK cells to microarray analyses. Fig. 5A shows that among the various cytokines, their receptors and signaling molecules represented on the chip, a single message encoding the IL-18 receptor showed a strong induction in post-activation NK cells. Increased IL-18 receptor expressions were also observed by FACS where mean fluorescence intensities were increased in NK1.1^+^/CD25^+^ cells after in vivo or in vitro activations 3.1- and 1.7-fold, respectively when compared with non-activated control NK cells (Fig. 5B). In addition, Western analysis showed increased IL-18 receptor protein levels when post-activation CD25-positive and non-activated NK cells were compared after expansions in IL-2 (Fig. 5B). We also determined IL-18 levels in the serum of B16-bearing mice and observed more than tenfold increases in a minority of mice (2 out of 19) while the remaining mice had levels similar to naïve controls (not shown). Together these data show that the IL-18 receptor expression was increased in murine post-activation NK cells even though expression differences appeared to be larger on the RNA when compared with the protein levels.

To investigate whether IL-18 receptor expression is also affected in human NK cell subsets, we analyzed its expression in blood cells from healthy donors. Fig. 5C shows increased IL-18 receptor levels when CD3^−^/CD56^br**ight**^ cells were compared with CD3^−^/CD56^dim^ NK cells (5.1-fold increase of its mean fluorescence intensity). Thus, murine post-activation and human CD56^br**ight**^ NK cells show an up-regulation of the IL-18 receptor.

## Discussion

Subsets of NK cells have been defined in humans and mice [4], [6], [7], [9], [10], [12], [36]. Here we investigate the relationship between NK cell activation and NK cell subsets. Asking whether known NK cell maturation stages correspond to the ability to respond to tumor activation we surprisingly observed that activation was detected only in the early subset of NK cells. Only CD27^high^/CD11b^low^ NK cells spontaneously degranulated and expressed CD25 after the in vivo exposure to the tumor environment. This suggests a lower activation threshold for early NK cells despite increased effector functions at later maturation stages [4]. A restriction of this subset to lyse tumor cells in vivo may have implications on NK cell-mediated anti-tumor therapy.

Tumor environments represent complex immunological sites harboring both monocytic and lymphocytic cells. Most immune cells in the tumor environment are believed to suppress immune reactions [37]. The presence of immune cells in the tumor environment may also influence the NK cell activation status. In our experiments however, in vitro exposures of NK cells to tumor lines grown in vitro could induce changes in cytotoxic ability and phenotypes that were similar to those seen after in vivo activation. Therefore, it appears likely that the changes that we observed in NK cells had been induced mainly by their encounter with tumor cells.

Tumor environments harbor substantial numbers of Tregs [38]. It has been reported that Tregs also limit the expansion of CD27^ high^/CD127^+^ NK cells [30]. Our data suggest that an exposure to tumor cells may activate the very same NK cell subpopulation. This suggests an equilibrium inside the tumor environment between NK cell-inhibitory and -activating signals. Tumor cell-induced activation may overcome Treg-mediated inhibition though the amplitude of NK cell activation may be controlled by Tregs. This may explain why NK cell activation was seen only when mice harbored large tumor burdens. It may also explain the increased resistance of *rag*-deficient mice to B16 (unpublished observation) since *rag* ^-/-^ mice lack Tregs. As a consequence, effects on tumor growth by Treg-targeting treatments such as anti-CTLA4 antibodies may also involve effects on NK cells.

It is unknown which tumor-NK cell interaction lead to NK cell activation. The loss of MHC class Ia expression appears to play a crucial role since its IFN-induced up-regulation in B16 or MC38 reduced their ability to activate and to be lysed by NK cells (not shown). We also compared NKR expression patterns between CD25^+^ and CD25^−^ NK cells after tumor-induced activations and observed biased expressions in CD25^+^ NK cells for several LY49 receptors (not shown) though no clear pattern emerged. In addition, tumor cells and/or their environment may express B7 family proteins that may contribute to the overall activation status of NK cells though again, no clear evidence was found for such a process.

IL-15 has been well described as a factor that is necessary for the survival and proliferation of mature NK cells. Both IL-15^-/-^ and IL-15Rα^-/-^ mice lack NK cells [39], [40], and neutralizing IL-15 activity *in vivo* causes a rapid depletion of NK cells [41]. It has also recently been reported that IL-15 signaling is required for NK cell priming [42]. Our data confirm that signaling through IL-15 supports the activation of NK cells. The most likely mode of action of this cytokine is a lowering of the activation threshold since culturing NK cells in IL-15 does not appear to affect NKR expression patterns (not shown). This activation appears to be a pre-requisite for the differentiation of NK cells into cells with regulatory function suggesting a role for IL-15 in NK cell maturation. This mechanism may be important when the use of IL-15 or its inhibition is considered for clinical purposes.

Post-activation NK cells were affected in their cytotoxicity, cytokine production and phenotype (Table S1). NK cells from naïve mice increased their cytotoxic activity when exposed to IL-2 in culture while post-activation NK cells failed to do so. This was accompanied by the lack of perforin and granzyme B. It appears most likely that post-activation NK cells had undergone a total degranulation that was followed by an inability to resynthesize perforin and granzyme B. Such an inability to partake in the crucial NK cell function of cytotoxicity suggests that post-activation NK cells may be directed towards immune-modulating functions.

We analyzed phenotypical changes in post-activation NK cells based on descriptions in human subsets [7], [31], [43]–[45]. Phenotypical changes were observed for CD16, CD25, CD62L, CD11c and the IL-18 receptor (Table S1). CD11c appears to represent an early activation marker whose expression is lost during IL-2 differentiation. Similar to the loss of cytotoxicity, the appearance of a population that lacks the Fc receptor CD16 suggests an inability to perform antibody-dependent cytotoxicity and may point to regulatory functions.

Analyses of cytokine production patterns in post-activation NK cells were also based on human subsets. When analyzed ex vivo, post-activation NK cells generated TNFα, IFNγ, IL-10 and GM-CSF. We note that the ability to generate cytokines was lost after the IL-2 culture. This may reflect an absence of signals in vitro that are necessary to maintain all NK cell functions. In vitro cultures in IL-2 cannot properly reflect in vivo conditions. Nevertheless, this cytokine culture does not only sustain but also change NK cell function such as cytotoxicity and their phenotypes.

In conclusion, tumor-induced and IL-15-supported murine NK cell activation appears to be limited to a subset of early NK cells. Such activations cause differentiation changes that result in low cytotoxicity and the ability to produce cytokines. Murine post-activation NK cells resemble human CD56^br**ight**^ NK cells in many aspects.

## Materials and Methods

### Ethics statements

All patients signed the informed consent for protocol 97-C-0143 “investigation of the human immune response in normal subjects and patients with disorders of the immune system and cancer.” Thus written and oral consent was obtained from all patients. The signed consent forms are part of the patients' permanent record. The ethics committee and the IRB of the National Cancer Institute NIH approved this consent procedure and specifically approved this study.

All mice were cared for under protocols approved by the NCI Animal Care and Use Committee that had specifically approved this study in accordance with National Institutes of Health (NIH) guidelines.

### Mice

C57BL/6 and C57BL/6-*Rag1* ^-/-^ mice were provided by The Jackson Laboratory. All mice used were females between 8 and 12 weeks of age. For tumor transplantations, one million of syngeneic B16 melanoma cells were injected i.v. in 200 µl PBS. NK cells were isolated from spleens 21 days later.

### Cytometry, ELISA, Cell Sorting and Culture

Antibodies directed against the following proteins that were used for cytometry were from BD Biosciences (CD56, human CD11c, CD16, IL-12Rβ2), Ebioscience (CD3, CD11b, CD20, CD27, CD90, CD127, NK1.1, CD25, granzyme B, perforin, CD62L, murine CD11c, CD122, CD127, 2B4, IFNγ, murine TNFα, human TNFα, GM-CSF, IL-10), Biolegend (PD1, CD107a) or from R&D Systems (murine and human IL-18R1). Blood cells were analyzed after removing erythrocytes via Ficoll-centrifugation. Erythrocytes were removed from spleen cell suspensions by lysis in ACK. For cytometry analyses, cells were blocked with a mixture of rat IgG1, IgG2a, IgG2b, mouse IgG1 and hamster IgG1 for 15 min at room temperature that was followed by a 30-min incubation on ice with the specific antibody. For biotinylated antibodies, an additional 15-min incubation on ice was done with streptavidin-PE-CY5 (BD Biosciences). The BD Cytofix/Cytoperm Kit was used to detect intracellular expression of granzyme B and perforin. Intracellular cytokines were detected after incubating cells for 5 h in the presence of brefeldin A according to the manufacturer's instructions (BD Biosciences). The ELISA kit to detect murine IL-18 was from Ebioscience, and serum analyses were done according to the manufacturer's instructions. NK cells were sorted from murine spleens or from human PBMCs using negative isolation microbeads (Miltenyi). Murine NK cell isolations resulted in greater than 95% of NK1.1^+^/CD3^−^/CD20^−^/CD122^hi^ cells with no detectable cells expressing CD3 or CD20 (not shown). NK cells were further separated into CD25^+^ and CD25^−^ populations by FACS. NK cells were cultured in RPMI 1640 supplemented with 10% Fetal Bovine Serum, 50 µM β–mercaptoethanol, antibiotics and 5 nM human IL-2 (Peprotech). For NK cell activation, freshly isolated spleen NK cells were co-cultured with equal numbers of B16 (kindly provided by Dr. Restifo, NIH) or EL-4 (ATCC TIB-39) cells for 6 h in medium that contained IL-2, IL-15 (Peprotech) or IL-15SA (kindly provided by R&D Systems) as indicated.

### Lysis Assay

For NK cell-mediated cytotoxicity we used sorted NK cells that had been cultured for 7 days in 5 nM IL-2. As target cells we used MC38 (kindly provided by Dr. Tagaya, NIH) and B16. Target cells (2.5*10^6^) were labeled with 1 mCi Chromium-51 (sodium chromate, Amersham) for 1 h at 37°C in 100% FBS and incubated for 4 h with effector cells at various effector:target ratios. Supernatants were transferred into 96-well plates (Wallac) and the radioactivity in the liquid phase was measured. Specific lysis was determined by using the formula: % lysis = 100 * [(mean experimental cpm - mean spontaneous cpm)/(mean maximum cpm - mean spontaneous cpm)]. The maximum release value was determined from target cells treated with 1% (v/v) Triton X-100 (Sigma).

### Western blot

Cell lysates were prepared from one million sorted and in vitro-expanded murine NK cells by lysis in 250 µl 4% SDS, 100 mM Tris, pH6.8, 20% glycerol. Lysates were sonicated, and 25 µl each were subjected to SDS-PAGE. Antibodies used for Western blotting were from R&D Systems (anti-IL-18R1), BD Biosciences (granzyme B and perforin) and from Sigma (β-actin).

### Microarray analysis

RNAs were isolated from ten million CD25^−^ and CD25^+^ NK cells after 7-day expansions in 5 nM IL-2 using Trizol (Invitrogen). Expressions of selected messages were analyzed with the Mouse Common Cytokines Gene Array according to the manufacturer's instructions (Superarray).

### Statistical analysis

Data are represented as means ± SD. Significance levels were compared using the Student's t test.

## Supporting Information

Table S1 **Comparison between murine post-activation and human CD56^bright^ NK cell subsets.**
(DOCX)
