# Supplementary material for: Loss of Cytotoxicity and Gain of Cytokine Production in Murine Tumor-Activated NK Cells
Source: PLoS One. 2014 Aug 7;9(8):e102793. doi: 10.1371/journal.pone.0102793 (PMC4125151; doi:10.1371/journal.pone.0102793)
Supplement: Table S1 — Comparison between murine post-activation and human CD56bright NK cell subsets. (DOCX) [file pone.0102793.s001.docx]

Table S1. Comparison between murine post-activation and human CD56^bright^ NK cell subsets

| Phenotypical changes | Murine | Human | Reference |
| --- | --- | --- | --- |
| CD56 | n.d. | high | [[31](#_ENREF_31)] |
| CD57 | n.d. | high | [[31](#_ENREF_31)] |
| CD27 | high | n.d. |  |
| CD90 | high | n.d. |  |
| CD127 | low | n.d. |  |
| CD11c | high | high/low | [[31](#_ENREF_31)] |
| CD16 | low | low | [[31](#_ENREF_31)] |
| CD25 | high | high | [[31](#_ENREF_31)] |
| CD62L | high | high | [[7](#_ENREF_7)] |
| IL-18 receptor | high | high | [[44](#_ENREF_44)] |

| Functional changes | Murine | Human | Reference |
| --- | --- | --- | --- |
| TNFα | + | + | [[43](#_ENREF_43)] |
| IFNγ | + | + | [[43](#_ENREF_43)] |
| IL-10 | + | + | [[43](#_ENREF_43)] |
| GM-CSF | + | + | [[43](#_ENREF_43)] |
| Cytotoxcity | low | low | [[45](#_ENREF_45)] |

n.d., not done
